# Influence of Comorbidity and Obesity on the Occurrence of Vascular Events in Obstructive Apnoea Treated with CPAP

**DOI:** 10.3390/nu16183071

**Published:** 2024-09-12

**Authors:** Inmaculada Jurado-Robles, Bernabé Jurado-Gámez, Nuria Feu Collado, Rafael Molina-Luque, Guillermo Molina-Recio

**Affiliations:** 1 Biosceiences and Agrifood Sciences Program, University of Córdoba, 14004 Córdoba, Spain; 2 GA03 Pneumology Maimonides Institute of Biomedical Research (IMIBIC), 14004 Córdoba, Spain; 3 Sleep Unit, Department of Respiratory Medicine, Reina Sofia University Hospital, 14004 Córdoba, Spain; nurifeco@gmail.com; 4 Nursing, Pharmacology and Physiotherapy Department, University of Córdoba, 14004 Córdoba, Spain; p72molur@uco.es (R.M.-L.); gmolina@uco.es (G.M.-R.); 5 Lifestyles, Innovation and Health (GA-16), Maimonides Biomedical Research Institute of Cordoba (IMIBIC), 14004 Córdoba, Spain

**Keywords:** CPAP, vascular event, obesity, sleep obstructive apnoea, nutritional status

## Abstract

Background: Obesity has increased cardiovascular morbidity and mortality. It is the leading risk factor for obstructive sleep apnoea (OSA). The relationship between obesity-OSA and vascular disease seems clear. There is no consensus on whether CPAP (continuous positive airway pressure) treatment prevents vascular events. Objective: The aim of this study was to determine the effect of comorbidity and obesity on the risk of vascular events in patients with OSA treated with CPAP. Method: This study was a prospective study of historical cohorts of adult patients with OSA and CPAP. The sample was 3017 patients. Descriptive, survival (Kaplan–Meier) and Cox regression analyses were performed, calculating crude and adjusted association relationships to explain the risk of vascular events. Results: A total of 1726 patients were obese, 782 were diabetics, and 1800 were hypertensive. The mean adherence was 6.2 (±1.8 h/day), and the mean follow-up time was 2603 days (±953.3). In the COX regression analysis, the event-related variables were baseline age (HR: 1.025: 1.012–1.037; *p* < 0.001), pre-treatment vascular event (HR; 2.530: 1.959–3.266; *p* < 0.001), hypertension (HR; 1.871: 1.187–2.672; *p* = 0.005) and abbreviated Charlson comorbidity index (HR; 1.289: 1.100–1.510; *p* = 0.002). Conclusions: The occurrence of vascular events in OSA patients on CPAP treatment is related to hypertension, having a vascular event before treatment, age at the start of CPAP use and abbreviated Charlson comorbidity index.

## 1. Introduction

Obesity is a chronic disease and a global public health challenge [1] as, together with overweight, it has become an epidemic with serious consequences for the health of the population [2]. In Spain, more than 50% of adults and almost 30% of children and adolescents are overweight or obese [3].

This situation is problematic because there is robust evidence of an association between obesity and increased risk of vascular morbidity and mortality [4]. Obesity leads to an increased incidence of major cardiovascular events, insulin resistance, endothelial dysfunction, sympathetic nervous system activation, increased vascular resistance and inflammatory and prothrombotic states [5]. In addition, it is associated with vascular disease [6]. In this regard, vascular risk is high or very high in type I obesity, very high in type II obesity and extremely high in type III obesity [7]. On the other hand, obesity has a direct impact on the development of dyslipidaemia, type II diabetes mellitus, hypertension and sleep-disordered breathing, which are, in turn, risk factors for the development of these diseases [8]. Ultimately, these pathologies are interrelated. Studies show that the prevalence of hypertension (HT) in patients with diabetes mellitus (DM) is as high as 80% and that having both conditions increases the risk of developing cardiovascular disease [9].

In addition, obesity is considered a crucial risk factor for obstructive sleep apnoea (OSA) [10]. Both pathologies, obesity and OSA, are currently considered vascular risk factors [11]. The data from the SLEEP-AHEAD study suggested that OSA was present in 86.6% of obese patients with type II diabetes [12]. It has also been shown that up to 70% of individuals with OSA are obese [13] and that the prevalence of OSA in obese or severely obese patients is almost twice as high as in normal-weight adults [14]. Furthermore, recent studies show that, as BMI increases, the risk of OSA increases, leading to a rise in the prevalence of AHI ≥ 15 of 3.6% in people with healthy weight compared to 56% in those with a BMI ≥ 40 among men aged 50–70 years [15].

Moreover, OSA affects 17% of women and 34% of men in the general population [16] and has a prevalence of 1–4% in children [17]. The Spanish Society of Pneumology and Thoracic Surgery (SEPAR) stated that “OSA is one of the most prevalent sleep disorders”, yet in Spain, only 25% of patients receive treatment [18]. Currently, international guidelines [19] have established the diagnostic criteria for OSA and have concluded that the most effective and evidence-based treatment for OSA is continuous positive airway pressure (CPAP) [20]. However, they also refer to the importance of a multidisciplinary therapeutic approach to the disease, emphasising hygienic-dietary measures and, especially, the nutritional approach to obesity. Weight loss (adipose tissue), lifestyle modification and increased physical exercise [21] are essential complementary measures in the treatment of OSA [22].

In addition, intermittent hypoxia occurring during the night in patients with OSA has also been associated with vascular disease [23], coronary atherosclerosis, increased platelet activity and myocardial ischaemia [24] and has, therefore, been considered an independent risk factor for these diseases [25]. The prevalence of OSA ranges from 40% to 80% in patients with hypertension, heart failure, coronary artery disease, pulmonary hypertension, atrial fibrillation and stroke [26].

In short, the close relationship between obesity-OSA, obesity-vascular diseases and OSA-vascular diseases seems evident [27]. For these reasons, the effects of OSA treatment with CPAP on OSA-related diseases have been studied. Some research has shown a decrease in the incidence of type II diabetes [28] and a reduction in blood pressure [29]. Other cohort studies have shown that CPAP treatment can reduce the incidence of vascular disease and the risk of mortality [30]. CPAP upper-airway stabilisation could benefit OSA symptoms and possibly vascular outcomes in some groups. However, there is no clear consensus on which patients may benefit most from its application [31]. Several randomised controlled trials have found negative results in terms of vascular prevention [32] as well as no improvement in biomarkers of inflammation and antioxidant activity [33]. Therefore, the modifying effect of CPAP treatment of OSA on vascular outcomes is highly controversial [34,35,36].

This controversy may be due to the small number of patients studied [28,29,30,33,35], the limited follow-up time of these patients [31,32,33,36] or the fact that different variables that may influence this effect have not been considered [30,31,32,34,35], limitations that we aim to overcome in this research.

Therefore, the main objective of our study is to analyse the role of obesity and comorbidity in the incidence of vascular events in patients undergoing CPAP treatment, understanding that vascular events are the ones diagnosed by medical staff following the International Classification of Diseases, tenth edition (ICD-10). In addition, as specific objectives, this study aims to establish the event type, the effect of events before treatment, the evolution of obesity measured through anthropometric parameters (BMI, abdominal circumference and neck circumference) and adherence to CPAP treatment.

## 2. Methodology

### 2.1. Design, Population and Sample

A historical (retrospective) cohort study was conducted of all adult patients at the Reina Sofia University Hospital in Cordoba diagnosed with OSA by home respiratory polygraphy or polysomnography [37,38] and prescribed CPAP treatment [39,40]. Patients whose treatment date was between 1 January 2000 and 31 December 2019 were included (*n* = 3017). The data collection period was from 1 December 2020 to 30 April 2023.

As inclusion criteria, patients had to be over 18 years of age and have been on CPAP treatment for at least one year with good compliance (use > 4 h/day). Patients who suffered from neuromuscular diseases with respiratory involvement and those who did not have the required data in the clinical history were excluded.

### 2.2. Variables and Data Collection

The dependent variable time was established as a function of the days elapsed between the start of CPAP treatment and the time the first vascular event occurred. In the case of no vascular event, the final date for calculating this variable was the day data collection ended (30 April 2023). A vascular event was considered to be any ischaemic cardiovascular or cerebrovascular disorder (stroke) for which the patient had to visit an emergency department. The diagnosis was made by medical staff following the International Classification of Diseases, tenth edition (ICD-10).

The independent variables were the following:

- Sociodemographics. Age (years) and sex (male and female).
- Anthropometrics. Weight (kg), height (cm), body mass index (BMI) (kg/m^2^), waist circumference (WC, cm), neck circumference (NC, cm), basal saturation (%), systolic (SST, mmHg) and diastolic (DBT, mmHg) blood pressure and the taper index [41] were calculated to estimate cardiovascular and metabolic risks.
- Clinical. Vascular event prior to OSA diagnosis and CPAP treatment (yes/no), abbreviated Charlson comorbidity index [42,43]: chronic obstructive pulmonary disease (COPD) (yes/no), heart failure or ischaemic heart disease (yes/no), neurocognitive deficit (yes/no), cerebrovascular disease (yes/no), arterial hypertension (yes/no), renal failure (yes/no), cancer (yes/no); in addition to whether the patient was a smoker (yes/no) and symptomatology collected in the Epworth Sleepiness Scale [44,45].
- Related to CPAP treatment: hours of compliance measured by the memory of each device.
- Pressure titration was initially calculated using the Miljeteig and Hoffstein formula [46] and subsequently using auto CPAP [47] as well as monitoring and correcting air leaks and possible side effects.
- Variables of the diagnostic study of polygraphy and polysomnography calculated per hour of recording in polygraphy or total sleep time in polysomnography. AHI: sum of apnoeas and hypopnoeas/hour; ODI: number of decreases ≥ 3% in O_2_ saturation (SpO_2_)/h; T90: percentage of time with SpO_2_ < 90% [20].

All variables were collected at the time of diagnosis of OSA (start of CPAP treatment) and at the time of the vascular event in those patients who experienced it. The data were recorded at the last check-up if no vascular event occurred.

### 2.3. Ethical and Legal Aspects

This study was carried out in compliance with the requirements established in Spanish legislation in the field of bioethical research, personal data protection and bioethics, updated through Organic Law 3/2018 of 5 December and respecting the fundamental principles established in the Declaration of Helsinki (1964), in the Council of Europe Convention on Human Rights and Biomedicine (1997) and the UNESCO Universal Declaration on the genome and human rights (1997). This research was approved by the Cordoba Research and Ethics Committee (Act nº 311, ref: 4736). All the patients gave their written informed consent.

### 2.4. Statistical Analysis

The calculation of our sample size, according to the methodology used for statistical analysis, for a proportion of 0.2 exposed and 0.8 unexposed, with a hazard ratio (HR) of 1.63 [33], estimated that the minimum number of vascular events required was 181. A descriptive analysis was performed, and summary tables were constructed with all patients, independently differentiating between those who had an event and those who did not have an event. Quantitative variables were presented as the mean and standard deviation, and qualitative variables were presented as absolute frequency and percentage. The survival of all patients was analysed with the Kaplan–Meier method, and multivariate Cox proportional hazards regression was used for each variable, estimating the crude and adjusted hazard ratio (HR) to determine their relationship with the event’s occurrence. For all statistical analyses, a probability of alpha error of less than 5% (*p* < 0.05) was accepted, and confidence intervals were calculated at 95%. SPSS (version 22.0) was used for the statistical analysis. The sample size was calculated using the sample size calculator [48].

## 3. Results

### 3.1. Incidence of Cardiovascular Events

**Figure 1:**
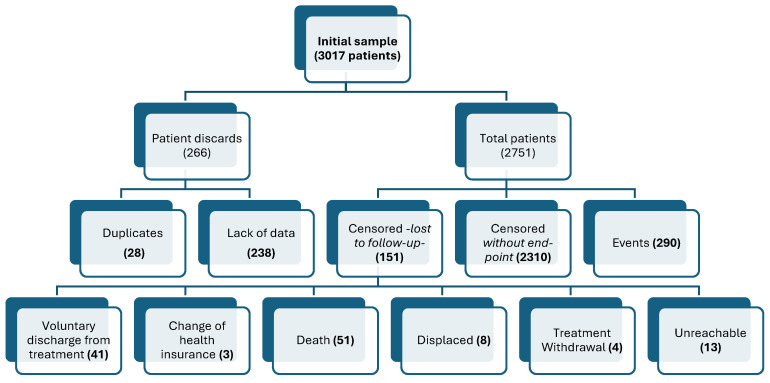
Patient flow diagram.

Of the 3017 patients in the initial sample, 28 were discarded due to duplicity in the system, and 238 were discarded due to missing data; therefore, the final sample size of this study was 2751 patients. On the other hand, 10.5% of these compliant patients had a vascular event. Out of a total of 290 events, 21 (7.2%) were cerebrovascular accident events (100% strokes), and 269 (92.8%) were cardiovascular events, with 4 aortic aneurysms, 4 angina pectoris, 34 unstable anginas, 1 trifascicular block, 2 bifascicular blocks, 6 grade I atrioventricular blocks, 2 grade II atrioventricular blocks, 4 complete atrioventricular blocks, 1 cor pulmonale, 37 acute myocardial infarctions, 4 aortic dissections, 8 aortic stenosis, 118 atrial fibrillations, 5 uricular flutter, 38 heart failures and 1 cardiac arrest (Figure 1).

### 3.2. Description of the Sample

Men constituted 72.3% of the sample. The mean age at baseline was 56 ± 11 years, and at the end of this study, the mean age was 63 ± 11 years, with the ages of the patients who had an event being 61 years at baseline and 65 years at the end. The mean BMI in both baseline and final data was overweight (33.8 ± 6.2 kg/m^2^ and 33.6 ± 6.1 kg/m^2^). This variable was higher in the patients who experienced events at baseline than in those who did not, but it was not significant (Table 1). Regarding concomitant diseases, 782 patients had diabetes, with 14.7% having suffered events. Hypertension was diagnosed in 1800 patients, 14% of whom had a history of a vascular event.

Concerning OSA severity, the AHI showed a mean of 50.1 (±24.9) and a T90 of 17.8 (±26.4). The mean CPAP use was 6.2 h per day (±1.8), thus with good adherence. The mean follow-up time of the patients was 2603.4 (±953.3) days of treatment, with a mean of 1224.8 (±809.6) days for the patients who experienced vascular events compared to 2765.9 (±826.8) days for those who did not. Furthermore, of the 1800 hypertensive patients, 650 (31.6%) were people with diabetes (Table 1).

### 3.3. Variables Related to the Occurrence of Vascular Events

Obesity (HR: 1.235; 95%CI 0.930–1641; *p* = 0.145) was not found to be a risk factor associated with vascular events after crude Cox regression. Of the comorbidity factors, end-diastolic blood pressure (HR: 0.984; 95%CI 0. 971–0.998; *p* = 0.023), cerebrovascular disease (HR: 3.799; 95%CI 2.436–5.924; *p* < 0.001), diabetes mellitus (HR: 1.715; 95%CI 1.355–2.170; *p* < 0.001), COPD (HR: 1.9041; 95%CI 1.332–2.723; *p* < 0.001), hypertension (HR: 3.676; 95%CI: 2.613–5.170; *p* < 0.001) and the abbreviated Charlson comorbidity value (HR: 1.750; 95%CI: 1.552–1.973; *p* < 0.001) showed this association. For the other variables studied, age at initiation of OSA treatment with CPAP (HR: 1.049; 95%CI: 1.037–1.061; *p* < 0.001) and history of vascular events before initiation of CPAP treatment (HR: 3.758; 95%CI: 2.947–4.793; *p* < 0.001) were also significant (Table 1).

### 3.4. Kaplan–Meier Survival Analysis

**Figure 2:**
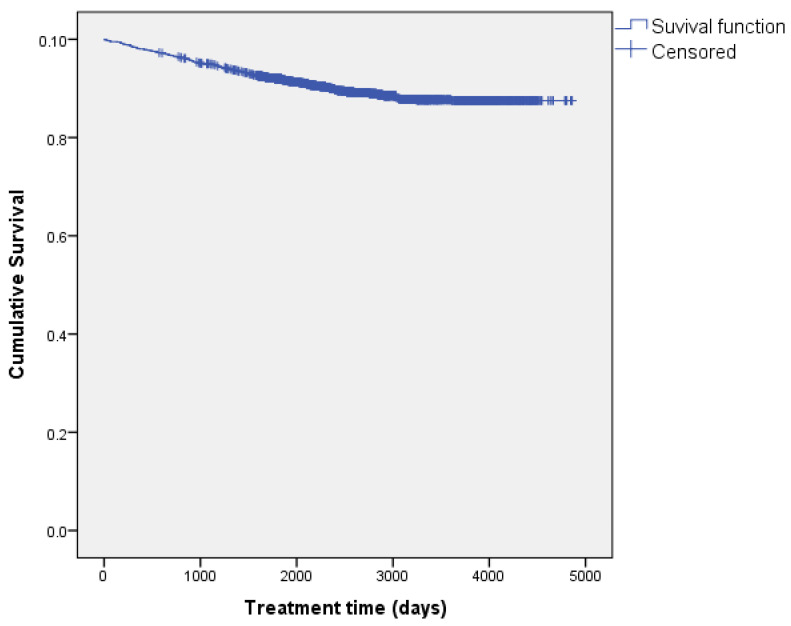
Kaplan–Meier curves showing cumulative survival.

**Figure 3:**
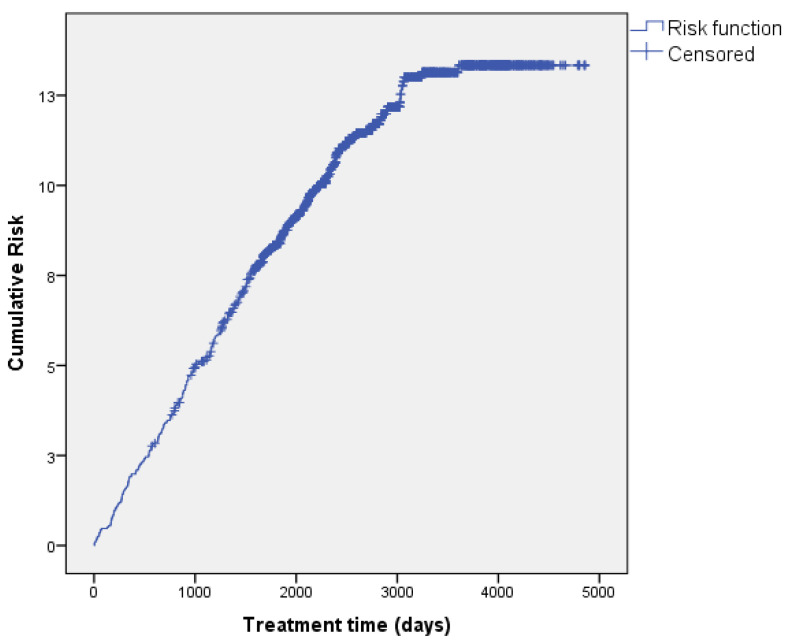
Line graph showing cumulative risk.

Fifty percent of the events (145) occurred in the first 3.2 years of treatment, and the cumulative survival estimate for that time was 94.7%. Most events (94.1%) occurred before 7.1 years, and the cumulative survival estimate at the end of this study was 87.5%, with the last event occurring at 9.9 years of follow-up. The median survival time estimate was 12.1 years (95%CI, 12–12.3). These data can be observed in the Kaplan–Meier curves, where the risk function increases over time until it stabilises above the median time, after which the patients show no events (Figure 2 and Figure 3).

### 3.5. Multivariate Survival Analysis

In the adjusted analysis, only age at initiation of OSA treatment with CPAP (HR: 1.025; 95%CI 1.012–1.037; *p* < 0.001), having suffered from a vascular event prior to initiation of treatment (HR: 2530; 95%CI 1.959–3.266; *p* < 0.001), suffering from high blood pressure (HR: 1.871; 95%CI 1.187–2.672; *p* = 0.005) and the abbreviated Charlson comorbidity index (HR: 1.289; 95%CI 1.100–1.510; *p* = 0.002) were related to vascular events. The model did not show collinearity problems (Table 2).

## 4. Discussion

A study of historical (retrospective) cohorts of OSA patients on CPAP treatment was conducted to determine the effect of obesity and other possible comorbidity factors on the risk of vascular events. The main finding of our study is that 10.5% of OSA patients suffered a vascular event, similar to a recent study (17%) [49]. However, in this work, all included subjects were previously diagnosed with coronary or cerebrovascular disease. Although our patients were well controlled on treatment and had optimal adherence to CPAP therapy [19], vascular events occurred in contrast to recent studies showing that good adherence to CPAP decreases the risk of these events [50]. This may be one of the reasons for the controversy of this study’s results [32], and it is hypothesised that having experienced an event prior to treatment is a risk factor for subsequent events.

Although the main objective of our study was to determine obesity as a risk factor for vascular events, as other researchers have highlighted [4,5,6,7,15], no significant differences were found between the patients who experienced an event and those who did not. This finding could be due to the high percentage of obese in our sample (69.3%), which is common in patients with OSA [13], and that, consequently, the mean BMI in both groups was 33.6 kg/m^2^. These results may have masked this association.

Another relevant aspect of the findings is the time trend shown by the occurrence of events. In our results, it is observed that the mean number of days of treatment in the subjects with vascular events is lower (1224.8 ± 809.6) than the mean number of days of treatment in those without events (2765.9 ± 826.8). These results are consistent with other studies that report a significant decrease in cardiovascular events in patients treated with CPAP after a mean follow-up of 2595 days [51]. On the other hand, the SAVE trial [52] did not find this significant risk reduction up to an average treatment duration of 1350 days, with a usage ratio of 3.3 h/d. This heterogeneity of results may be related to the difference in mean CPAP use, which in our trial was 6.2 ± 1.8. This finding may lead us to consider the association between late diagnosis and the onset of severe untreated disease.

Age has been described as a non-modifiable risk factor for cardiovascular disease [53,54]. However, the value of this variable in our patients did not significantly impact the event’s outcome. In contrast, age at baseline (when they started CPAP treatment for OSA) showed a significant effect on the occurrence of vascular complications. Specifically, the patients who experienced events started treatment at an older age than those who did not (HR: 1.049). The findings seem to indicate the need for early diagnosis of OSA in patients and the establishment of protocols to cover a larger population, as the disease is underdiagnosed today, which may mean that treatment is established after years of suffering from the disease, which seems to worsen the prognosis [55].

On the other hand, as other studies show, having some vascular event prior to CPAP treatment, regardless of its severity, significantly influences the development of a subsequent event. Of the 2751 patients in our sample, 394 had such a history, and 25.4% had a repeat event after initiation of treatment. This result is in line with another publication, which concluded that CPAP has no positive effect on the prevention of major cardiovascular events in patients with OSA and previous acute coronary syndrome and reported an incidence of 26% during a follow-up of 3.3 years (1222.7 days) [56]. Other publications show that suppression of respiratory apnoeas with CPAP and consequent correction of nocturnal intermittent hypoxia in patients with established cardiovascular disease do not necessarily lead to remission of these diseases [57]. Therefore, it is vital to emphasise the importance of early diagnosis of OSA, which opens an exciting scenario for primary prevention.

On the other hand, our study shows that the Charlson comorbidity index behaves as a risk factor for vascular events, with results that coincide with those of other authors [58]. In this sense, there is evidence that HBP is the concomitant disease that appears as one of the most influential risk factors in vascular complications [59]. Viteri et al. (2017) corroborated this association. They indicated the relevance of carrying out HBP screening programmes in the high-risk population (low- and middle-income patients, obese patients and patients with diabetes) to reduce the complications associated with it [60]. These findings coincide with those of our research.

The risk associated with type 2 diabetes mellitus in events such as acute myocardial infarction, angina pectoris and coronary revascularisation has been demonstrated [61], as well as stroke and fatal cardiovascular events [62]. However, even though this variable appeared significant in the bivariate analysis of our study, it did not show this association in the adjusted multivariate model. In the case of the sample studied, we found that most of the people with diabetes were hypertensive. This situation may have masked this association, but comparing it with previous studies was impossible since none measured HBP as a variable.

## 5. Limitations

In addition to the weaknesses of a retrospective (historical) cohort [63], the main limitation is data collection due to a lack of clinical information. This fact meant that we had to discard part of our initial sample. It would have been desirable to include other anthropometric data to assess nutritional status, such as body fat percentage or muscle mass. However, since few records included these, they were not included in this study.

The incidence of vascular events in our study was 10.5%, so it was impossible to estimate the median survival rate, and we were only able to obtain data on the mean, with the limitations that this implies in terms of the different exposure times of each participant. On the other hand, these data were unavoidable, since in other studies as in ours, the incidence is similar and in no case reaches 50%.

It should be added that our study is unicentric, with the cultural characteristics of the patients, healthy habits and dietary habits different from other populations, so the results could be extrapolated to a population with characteristics similar to our sample.

On the other hand, a strength that should be highlighted is that our study was conducted in the same sleep laboratory the diagnosis was made with the gold standard technique (polysomnography) or with cardiorespiratory polygraphy, a valid technique for the diagnosis of OSA and validated in our laboratory [36], and the treatment and follow-up were carried out exclusively according to clinical practice guidelines. In addition, the sample number was large, well above the minimum sample number calculated for this study.

## 6. Conclusions

We conclude that the occurrence of vascular events in patients with OSA on CPAP treatment is related to hypertension, having a vascular event before the start of treatment, age at the start of CPAP use and the abbreviated Charlson comorbidity index. The results highlight the importance of early diagnosis of OSA, the establishment of treatment and optimal adherence to therapy and the importance of reaching the entire affected population. Therefore, further studies should focus exclusively on the study of the characteristics of patients affected by these events, the variables related to their occurrence and the interactions between them.

## Figures and Tables

**Table 1:** Characteristics of the sample at the start of this study and according to the occurrence or non-occurrence of the vascular event.

		Total (2751)	With Event (290)	Without Event (2461)	HR	95%CI	*p*
Age Initial (years)		56 ± 11	61 ± 10	55 ± 11	1.049	1.037–1.061	**<0.001**
Sex, *n* (%)						0.757–1.262	0.859
	Male	1990 (72.3%)	208 (10.4%)	1782 (89.6%)	0.977		
	Female	761 (27.7%)	82 (10.8%)	679 (89.2%)	1 (ref)		
Previous event *n* (%)		394 (14.3%)	100 (25.4%)	294 (74.6%)	3.758	2.947–4.793	**<0.001**
DM *n* (%)		782 (28.4%)	115 (14.7%)	667 (85.3%)	1.715	1.355–2.170	**<0.001**
COPD *n* (%)		181 (6.6%)	34 (18.8%)	147 (81.2%)	1.904	1.332–2.723	**<0.001**
Dementia *n* (%)		2 (0.1%)	0 (0%)	2 (100%)	0.050	0.000–5,679,451.284	0.751
HBP *n* (%)		1800 (65.4%)	252 (14%)	1548 (86%)	3.676	2.613–5.170	**<0.001**
Dialysis *n* (%)		10 (0.4%)	3 (30%)	7 (70%)	2.727	0.874–8.505	0.084
Cancer *n* (%)		217 (7.9%)	30 (13.8%)	187 (86.2%)	1.347	0.923–1.966	0.122
Charlson Comorbidity *n* (score)		1.1 ± 0.9	1.6 ± 0.8	1.1 ± 0.9	1.750	1.552–1.973	**<0.001**
Smoker *n* (%)		491 (17.8%)	51 (10.4%)	440 (89.6%)	0.959	0.708–1.397	0.784
Alcohol consumption *n* (%)		107 (3.9%)	11 (10.3%)	96 (89.7%)	0.891	0.487–1.627	0.706
AHI (Events/h)		50.1 ± 24.9	48.2 ± 24.6	50.2 ± 24.9	0.996	0.990–1.001	0.128
ODI (Events/h)		50.1 ± 29.3	48.2 ± 25.7	60.3 ± 29.6	0.996	0.991–1.001	0.122
T90 (%)		17.7 ± 26.4	20.6 ± 23.9	17.4 ± 26.6	1.002	0.999–1.005	0.133
Prior ESS (score)		11.6 ± 4.2	11.2 ± 4.3	11.6 ± 4.2	0.981	0.948–1.014	0.255
SpO_2_ Basal (%)		96.7 ± 1.5	96.6 ± 1.6	96.8 ± 1.5	0.943	0.863–1.031	0.198
Initial BMI (kg/m^2^)		33.8 ± 6.2	34.3 ± 6	33.7 ± 6.2	1.013	0.992–1.033	0.224
Initial obesity *n* (%)		1726 (62.7%)	174 (10.1%)	1552 (89.9%)	1.283	0.949–1.734	0.105
Initial TI (index)		1.4 ± 0.1	1.4 ± 0.1	1.8 ± 0.1	2.101	0.340–12.983	0.424
Initial waist circumference (cm)		111.8 ± 13.7	112.9 ± 13	111.7 ± 13.9	1.006	0.992–1.020	0.410
Initial neck circumference (cm)		39.1 ± 5.8	39.9 ± 5.8	39 ± 5.8	1.022	0.962–1.085	0.486
Initial SBP (mmHg)		133.3 ± 14.4	132.7 ± 14	133.4 ± 14.4	0.998	0.988–1.008	0.699
Initial DBP (mmHg)		76.6 ± 11.5	75.2 ± 11	76.7 ± 11.5	0.990	0.977–1.003	0.127
Age Last (years)		63.8 ± 11.3	65.2 ± 10.6	63.6 ± 11.4	1.009	0.999–1.020	0.084
BMI Last (kg/m^2^)		33.6 ± 6.1	34 ± 6.1	33.5 ± 6.1	1.011	0.991–1.031	0.278
Obesity Last *n* (%)		1907 (69.3%)	187 (9.8%)	1720 (90.2%)	1.235	0.930–1.641	0.145
Final SBP (mmHg)		137 ± 16	135.2 ± 17.2	136.8 ± 15.8	0.993	0.985–1.002	0.108
Final DBP (mmHg)		78.1 ± 9.9	76.6 ± 10.7	78.3 ± 9.8	0.984	0.971–0.998	**0.023**
ESS Last (score)		2.7 ± 3.6	2.5 ± 3.6	2.8 ± 3.6	0.981	0.948–1.015	0.275
CPAP Pressure (mm Hg)		8.2 ± 1.2	8.1 ± 0.9	8.2 ± 1.2	0.986	0.889–1.094	0.797
CPAP Rate (hours/day)		6.2 ± 1.8	6.3 ± 2.2	6.1 ± 1.8	1.038	0.973–1.107	0.261

Description of means of total patients, patients with CVD and patients without CVD. COX regression for independent variables. DM: diabetes mellitus; HBP: arterial hypertension; AHI: sleep apnoea hypopnoea index divided by recording time (polygraph) or sleep (polysomnography); ODI: number of decreases ≥ 3%/h in SpO_2_; T90: time with SpO_2_ < 90%; SBP: systolic blood pressure; DBP: diastolic blood pressure; BMI: body mass index; TI: taper index; Rate: use in hours per day of CPAP equipment. Mean treatment time of treatment of the sample (2603.43 ± 953.35) patients; with events (1224.78 ± 809.64) and without events (2765.89 ± 826.78). ESS, Epworth Sleepiness Scale. Bold numbers represent significant differences.

**Table 2:** Variables related to the occurrence of events in OSA patients treated with CPAP.

Variables	With Event (290)	Without Event (2461)	HR	95%CI	*p*
Age Initial (years)	55.5 ± 11.2	61.3 ± 10.3	1.025	1.012–1.037	**<0.001**
Previous event (%)					
No	2167 (91.9%)	190 (8.1%)			
Yes	294 (74.6%)	100 (25.4%)	2.530	1.959–3.266	**<0.001**
HBP (%)					
No	913 (96%)	38 (4%)			
Yes	1548 (86%)	252 (14%)	1.781	1.187–2.672	**0.005**
Charlson Comorbidity Index	1.1 ± 0.9	1.57 ± 0.8	1.289	1.100–1.510	**0.002**

Cox regression; HBP: hypertension. The model has no collinearity problems. Bold numbers represent significant differences.

## Data Availability

The anonymised database will be made available to researchers upon reasonable request. Data cannot be shared publicly due to patient data protection law, as well as being part of another ongoing study.

## Abbreviations

OSA	Obstructive Sleep Apnoea
AHI	Apnoea and Hypopnoea Index
EV	Vascular Diseases
HBP	High Blood Pressure
CPAP	Continuous Positive Airway Pressure
BMI	Body Mass Index
PG	Respiratory Polygraphy
PSG	Polysomnography
T90%	Percentage of time with peripheral oxygen saturation ≤ 90%
ID3	Oxygen Desaturation Index or number of drops in peripheral oxygen saturation ≥ 3%/h of recording
HR	Heart Rate
SpO_2_	Peripheral Oxygen Saturation
